# Toward Measuring Cognitive Endurance Capacity: A Proof-of-Principle Study in a Healthy Population

**DOI:** 10.1007/s10926-025-10346-8

**Published:** 2026-01-13

**Authors:** Fabienne I. M. van Vliet, Birgit H. P. M. Donker-Cools, Gert J. Geurtsen, Rudolf W. H. M. Ponds, Frederieke G. Schaafsma

**Affiliations:** 1 Department of Public and Occupational Health, Amsterdam Public Health Research Institute, Amsterdam University Medical Centres, Amsterdam, The Netherlands; 2 Department of Medical Psychology, Location AMC and VUmc, Amsterdam University Medical Centres, Amsterdam, The Netherlands; 3 Department of Public and Occupational Health: Research Centre for Insurance Medicine (KCVG), AmsterdamUniversity Medical Centres (AmsterdamUMC), Amsterdam, The Netherlands

**Keywords:** Cognitive endurance, Cognition, Neuropsychological assessment, Mental and cognitive fatigue

## Abstract

**Purpose:**

Occupational health professionals increasingly encounter workers who complain about a decrease in cognitive endurance, yet standardized tools to objectively assess this decrease are lacking. Cognitive endurance, the ability to sustain cognitive performance during prolonged mental effort, is critical for sustainable employability.

**Methods:**

In this proof-of-principle study, 101 healthy working adults (M_age = 35.7, SD = 13.6) completed neuropsychological assessments and fatigue questionnaires at the start and end of a regular workday. Cognitive endurance was operationalized as within-day change scores across six cognitive domains. Analyses included paired t-tests, Wilcoxon signed-rank tests, Spearman correlations, and multiple regression.

**Results:**

Over the course of a workday, cognitive performance improved in language, attention/executive functioning, and information processing speed (all p < 0.001), despite a significant increase in subjective fatigue (Z = –6.34, p < 0.001). Nearly half of the neuropsychological tests showed improvement. Fatigue measures explained only a small part of the variance in language change (R^2^ = 0.122), and no individual fatigue predictors were significant in that model. Higher subjective fatigue was associated with less improvement in processing speed (p = 0.040).

**Conclusion:**

Cognitive performance remained stable or improved over the course of the day, even as subjective fatigue levels increased. Fatigue measures showed limited predictive value for changes in cognitive outcomes, with only subjective fatigue significantly related to performance in one domain. These findings suggest that in healthy working adults, subjective complaints alone do not reliably indicate reduced cognitive functioning. Incorporating objective cognitive data may therefore be important when assessing endurance capacity and informing occupational health evaluations.

**Supplementary Information:**

The online version contains supplementary material available at https://doi.org/10.1007/s10926-025-10346-8.

## Introduction

Mental endurance capacity refers to the ability to maintain cognitive performance during extended periods of mental effort. It is increasingly recognized as essential for sustained work participation among individuals with chronic health conditions [1]. The relevance of this concept has grown, especially in the context of return-to-work and work sustainability. Chronic health problems substantially increase the risk of loss of paid employment and reduced work participation [2]. This is partly explained by fatigue and reduced endurance, which may limit individuals’ ability to sustain full participation during the workday [3, 4]. Individuals with chronic health conditions also frequently experience cognitive difficulties, such as problems with sustained attention, mental fatigue, or slowed thinking, which may or may not be accompanied by measurable impairment [5–7]. These observations highlight the importance of distinguishing between general fatigue and the ability to sustain cognitive performance. While the term ‘mental endurance’ has been commonly used in earlier literature [1], we adopt ‘cognitive endurance’ to emphasize its task-specific, neurocognitive nature.

Work endurance, more broadly defined by Boersema et al. [8], includes both physical and cognitive capacity to perform work over a period, typically expressed in hours per day or week. This “period” is individual and does not always imply full-time work. Even part-time hours may exceed a person’s endurance threshold, particularly in the presence of persistent cognitive complaints [8]. In the Netherlands, nearly 40% of workers with chronic health conditions are unable to work full-time, mainly because of reduced energy levels and (mental) fatigue [3], with higher prevalence rates in older workers (45.1% of those aged 55–64 and 51% of those aged 64–74) [9, 10].

Occupational health professionals (OHPs) increasingly assess not only whether individuals can perform cognitively demanding tasks, but also whether this performance is sustainable over time [8]. Cognitive endurance, impacting both capacity and duration, is key in return-to-work and workload decisions. Difficulties with endurance occur not only in neurological conditions but also in chronic somatic illnesses like cardiovascular and musculoskeletal disorders, often involving mental slowing, reduced concentration, and fatigue, even without brain injury [7]. Such complaints are common in cognitively demanding jobs and predict long-term disability and reduced work participation, even in the absence of objective impairment [11].Although related concepts such as cognitive load, sustained attention, and mental fatigue are discussed in the literature, they do not fully capture the dynamic and time-dependent nature of maintaining cognitive performance under prolonged demands.

Drawing on Cremer’s framework [1, 12], we define “cognitive endurance” as the capacity to sustain stable, efficient performance despite increasing cognitive load or time on task, a definition central to this study. Cognitive endurance decline may manifest as reduced accuracy or attentional control, particularly during tasks requiring sustained focus, high cognitive load, or problem-solving [5]. These effects can impact next-day performance after cognitively intensive work [13, 14].

Similarly, we use the term cognitive fatigue to refer to performance-related declines in attention or executive functioning, distinguishing it from more general forms of emotional or psychological exhaustion [6, 15]. A reduction in such sustained performance, often termed cognitive fatigue, is considered a key mechanism underlying decreased endurance [6, 16] and may result not only from diminished cognitive capacity but also from reduced motivation and error monitoring under fatigue [17]. Holtzer et al. [18] described it as a disruption in executive attention during prolonged effort, while DeLuca [6] showed that performance could deteriorate not only after intense exertion but also during extended mental activity. Kluger et al. [15] refined this by distinguishing between subjective fatigue (the perceived sense of exhaustion) and fatigability (a measurable decline in performance). This distinction remains highly relevant in occupational health, where reported complaints often do not align with objective outcomes. Wylie et al. [16] distinguished between trait fatigue, a general vulnerability, and state fatigue, which fluctuates during the day. Both were found to be important in evaluating performance sustainability. Few tools objectively measure within-day cognitive performance decline in occupational settings. The Mental Endurance Capacity Assessment by Cremer [1] combines neuropsychological tests, self-reports, and behavioral observation, but lacks standardization, normative data, and formal validation [8], limiting its use in real-life assessments. As a result, OHPs have limited access to reliable methods for assessing cognitive endurance at work. Current assessments mostly rely on neuropsychological tests (NPAs) and self-report questionnaires. While NPAs validly detect cognitive impairments [19], they are limited to single time points. Self-reports reflect fatigue but are sensitive to context and introspective bias [20, 21].

This study was designed as a proof-of-principle to explore whether cognitive endurance could be objectively assessed through observable performance changes during a regular workday. Given the current lack of instruments sensitive to within-day variability, we tested whether repeated cognitive assessments at the start and end of a typical working day could reliably capture subtle, yet meaningful fluctuations. Previous research has shown that repeated exposure to cognitive tasks can lead to modest improvements in performance due to practice effects, such as task familiarity and procedural learning, even over short time intervals [22]. These expected gains in healthy individuals may mask subtle fatigue-related changes, making it particularly important to examine both subjective and objective measures over the course of the day. Using a healthy working population, we aimed to establish baseline patterns of performance change under naturalistic conditions before applying this approach to clinical groups.

We hypothesized that subjective fatigue would increase across the day, while objective cognitive performance would remain stable or slightly improve. This expectation was based on participants being cognitively healthy working adults, for whom test performance was unlikely to decline to clinically relevant levels within a single workday. Practice effects from repeated testing, such as improved task familiarity and efficiency, were also expected to contribute to stable or improved scores. Small declines or reduced gains might occur in tasks involving sustained attention or executive control, but such changes were expected to remain within a normal functional range, that is, not indicative of cognitive impairment or abnormal functioning. By comparing subjective fatigue with objective performance change, we aimed to explore the feasibility of using repeated cognitive testing to assess cognitive endurance in real-world work settings.

## Methods

### Study Design

**Fig. 1:**
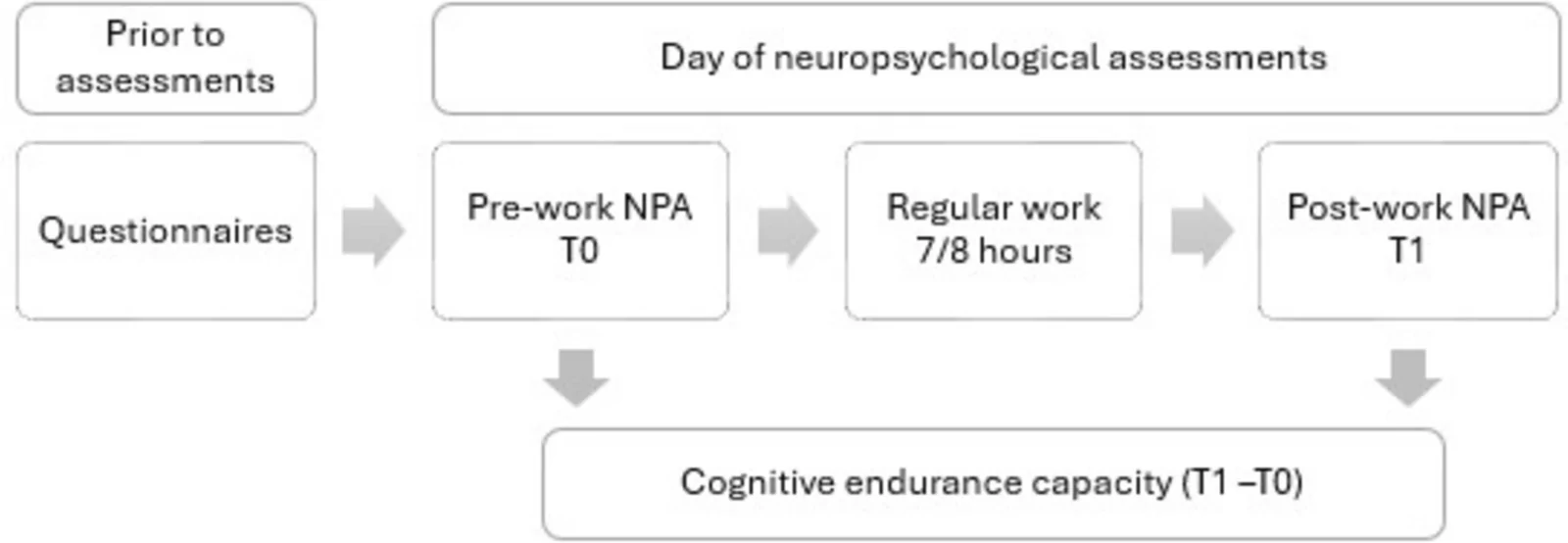
Overview of the constructed study design for measuring cognitive endurance capacity. Note. *NPA* neuropsychological assessment; *T0* pre-work assessment, *T1* post-work assessment

To assess the impact of a workday on cognitive performance, a pre-post within-subject design was employed. Participants completed neuropsychological assessments (NPA) twice during a single workday: once at the start and once at the end of their workday. Parallel test versions were used when available to minimize learning effects. Each NPA session lasted approximately 90 min. No separate familiarization session was conducted before the pre-work assessment, as the aim was to capture participants’ natural cognitive performance under regular working-day conditions. Participants also completed self-report questionnaires one week prior to the NPA and again during the assessment day. Cognitive endurance is operationalized as the difference (T1 – T0) in test performance between the post-work (T1) and pre-work (T0) assessments. Subjective trait and state fatigue were assessed using self-report questionnaires administered prior to and during the assessment day. See Fig. 1 for an overview of the schedule.

### Sample

Between March 2022 and October 2024, 101 healthy working individuals were recruited via convenience sampling. Recruitment took place via flyers and the researchers’ personal and professional networks. Informed consent was obtained from all participants prior to their inclusion in the study. Each participant was tested on one regular working day that matched their usual schedule. Testing days were not standardized or randomized across weekdays or seasons. Everyone participated voluntarily and anonymously and was allowed to withdraw at any time. The Medical Ethics Committee of the Academic Medical Centre Amsterdam granted a dispensation from full ethical review (ref: W22_408 #22.483). The assessments and data handling were carried out in accordance with Dutch legislation and the local guidelines of Amsterdam UMC.

The term “healthy working adults” was used functionally, referring to individuals who were employed, not on sick leave, and able to perform their regular work tasks. This definition follows a dynamic view of health as the ability to adapt and self-manage despite minor or stable health conditions [23, 24].

### Inclusion and Exclusion Criteria

Participants were eligible for inclusion if they met all the following criteria:

- Aged 18–68 years
- Employed or studying ≥ 24 h/week (students: ≥ 8 h paid work/week, and working on the day of assessment)
- Fluent in Dutch (spoken and written)
- Able and willing to complete neuropsychological testing

Participants were excluded if they met any of the following criteria:

- Currently on sick leave
- Diagnosed with a neurological disorder or cognitive impairment

### Instruments

#### Main Study Parameter

##### Neuropsychological Assessment (NPA)

The neuropsychological battery included 14 standardized verbal and nonverbal tasks, administered over approximately 90 min. These tasks measured six cognitive domains: attention and executive functions, language, memory, visuospatial abilities, information processing speed, and reaction speed. Table 1 provides an overview of the neuropsychological tests used. All assessments were conducted or supervised by licensed clinical neuropsychologists to ensure data quality and reliability.

**Table 1:** Overview of neuropsychological tests grouped by cognitive domain

Cognitive domain	Neuropsychological test
Language	Semantic Fluency (Animals, Occupations), Phonological Fluency (DAT, KOM)
Memory	Auditory Verbal Learning Test (AVLT), Rivermead Behavioral Memory Test (RBMT)
Attention & Executive Functions	WAIS-IV Digit Span, Stroop (Card III), Trail Making Test Part B (TMT-B), D2 Test (total items processed corrected for errors (TN-F), total number of errors (F), concentration performance (CP) , Tower of London (TOL)
Information Processing Speed	Stroop (Cards I & II), Trail Making Test Part A (TMT-A), WAIS-IV Coding, D2 (Total number of items processed (TN), variation in speed (VT)). , TOL (Problem-Solving Time)
Reaction Speed	Vienna Test System (RT S1 & S3: reaction time, motor speed, errors)
Visuospatial Abilities	Groninger Intelligence Test 2 (GIT-2): Perceptual Puzzle
Performance Validity	Amsterdam Short-Term Memory Test (ASTM)

**Note:** Parallel versions were used for: Phonological Fluency, AVLT, RBMT, TMT, and ASTM

Most tests were scored using normative data from the ANDI database [25], providing T-scores adjusted for age and/or education (M = 50, SD = 10). Tests not covered by ANDI, including the Vienna Test System (VTS) and D2, were scored using their original manuals. Test assignment to cognitive domains followed the ANDI framework and was supplemented with clinical reasoning where needed, for example: the Tower of London (executive functioning) and VTS reaction speed (VTS-RT). Where no direct mapping existed, tests were grouped based on primary cognitive demands [26]. Composite scores per domain were calculated by averaging the standardized scores; see Table 1.

##### Questionnaires

One week before testing, participants completed a series of self-report questionnaires to assess mental health status, trait fatigue, psychosocial work factors, and state fatigue.

Mental health status over the preceding weeks was assessed using the General Health Questionnaire (GHQ-12), consisting of 12 items (range 0–12) [27]. A score of ≥ 2 indicated potential mental health problems [27–29]. General Health Questionnaire scores were used descriptively and not as exclusion or control criteria.

Trait and state fatigue were assessed at different time points. Subjective trait fatigue was assessed using the Dutch Checklist Individual Strength (CIS), comprising 20 items across four subscales: fatigue, concentration, motivation, and activity level [30]. The total score (range 20–140; > 76 = severe fatigue) reflects overall fatigue-related functioning. The primary subscale, subjective fatigue (8 items, range 8–56), captures perceived fatigue severity and was categorized as normal (< 27), increased (27–35), or severely increased (> 35) [21, 30]. Subjective state fatigue was assessed at the start of each NPA using a Numeric Rating Scale (NRS) ranging from 10 (very tired) to 0 (not tired at all).

Psychosocial work factors were evaluated using the Dutch version of the Job Content Questionnaire (JCQ), based on the demand–control model [31]. This 35-item measure included scales for skill discretion, decision authority, psychological demands, and social support, scored on 4-point Likert scales with Dutch norm values used for interpretation. Norm values provided a benchmark to interpret scores by comparing individual or group results to the average scores in the population [32].

#### Other Study Parameters

De-identified participant data, collected through self-report, included age, gender, presence of chronic somatic conditions (yes/no), DSM diagnosis (yes/no), medication use (yes/no), and educational level (based on the Dutch Verhage scale: low [1–4], middle [5], and high [6–7]) [33]. Work characteristics included employment or student status, type of work (physical, mental, or both), and number of working hours per week.

### Statistical Software

Data from the online questionnaires were gathered using Castor EDC [34]. All data analyses were conducted with the SPSS (IBM SPSS 28) [35].

### Power Analysis

An a priori power analysis (G*Power 3.1) [36] was conducted to determine the required sample size. As no prior data on cognitive endurance effects were available, a conservative small effect size (*Cohen’s d* = 0.25) was chosen. With α = 0.05 and power = 0.70, a minimum sample size of N = 101 was required for paired t-tests.

### Descriptive and Preliminary Analyses

Descriptive statistics were computed for cognitive test scores, fatigue measures, and job characteristics. Data distribution was evaluated visually (histograms, boxplots, Q–Q plots) and statistically (skewness, kurtosis, and the Kolmogorov–Smirnov test). The Kolmogorov–Smirnov test, which is appropriate for larger samples (*n* > 50), indicated that 21 out of 30 test variables deviated from normality. As a result, non-parametric tests were applied in subsequent analyses to ensure methodological consistency.

### Main Analyses

Cognitive endurance was defined as the difference in performance between T0 (morning) and T1 (afternoon). For each test, within-day change was assessed using the Wilcoxon signed-rank Test. A Bonferroni correction was applied to adjust for multiple comparisons (α = 0.00167). Effect sizes (r) were calculated for each Wilcoxon test using the formula $r=\frac{z}{\sqrt{N}}$​, where Z is the Wilcoxon test statistic and N the number of non-tied observations. Effect sizes were interpreted as small (r ≥ 0.1), medium (r ≥ 0.3), and large (r ≥ 0.5) effects. This ‘r’ represents a standardized effect size analogous to Cohen’s criteria, but should not be interpreted as a correlation coefficient [37].

At the domain level (e.g., language, memory, attention/executive functioning, processing speed, reaction speed, visuospatial ability), mean scores for T0 and T1 were compared. Normality of domain scores was assessed using the Kolmogorov–Smirnov test. Paired t-tests were used where assumptions were met; otherwise, the Wilcoxon test was applied (e.g., for visuospatial ability). Because these six domain scores were a priori defined and conceptually correlated, no multiple-comparison correction was applied at this level.

### Correlation and Regression Analyses

Due to non-normal distributions, Spearman’s rank correlation was used to examine the associations between cognitive change scores, fatigue measures, and work-related variables. To explore whether fatigue predicted cognitive performance change, multiple linear regression analyses were performed. Assumptions for regression, linearity, normality, homoscedasticity, and absence of multicollinearity were met (Durbin-Watson ≈ 2.0; VIF < 5). Residuals were evaluated using Q–Q plots and the Shapiro–Wilk test.

For each cognitive domain, the dependent variable was the difference score (T1–T0), reflecting within-day change. We conducted simultaneous multiple regression analyses to assess the unique and combined contributions of fatigue-related predictors, while controlling for overlap between trait, subjective, and state fatigue. This approach also minimized the risk of false positives due to multiple testing. Three fatigue predictors were entered simultaneously: pre-work fatigue (NRS), trait fatigue (CIS total score), and subjective fatigue (CIS subscale), selected a priori to capture both state and trait dimensions. Full descriptive and inferential statistics for all neuropsychological tests, fatigue measures, correlations, and regression models are provided in the Online Resources 1–8.

## Results

### Sample and Baseline Characteristics

The study included 101 healthy working adults (57 females, 44 males), aged 19 to 68 years (M = 35.7, SD = 13.6), of whom 81.2% had completed higher professional or university education. A minority reported a somatic disorder (9.9%), such as hypothyroidism, Crohn’s disease, or type 1 diabetes, or a psychiatric diagnosis (15.8%), including ADHD and anxiety disorders. Additionally, 23.8% used medication. Notably, 33.7% scored above the General Health Questionnaire cut-off, suggesting possible psychological distress. Full demographic and questionnaire data are presented in Online Resource 1 and 2.

**Fig. 2:**
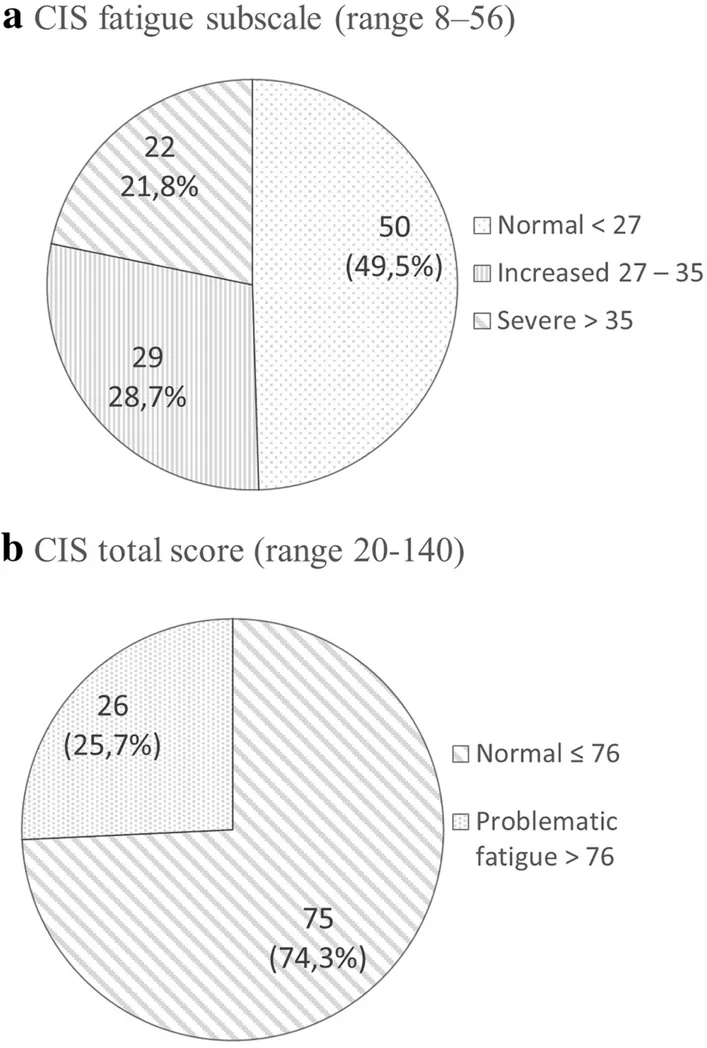
**a** and **b** Trait fatigue measurements

On the CIS, 25.7% of participants scored above the clinical threshold (≥ 76) on the total score, indicating problematic trait fatigue (Fig. 2a). On the subjective fatigue subscale, which specifically reflects perceived fatigue severity, 49.5% reported normal fatigue (< 27), 28.7% increased (27–35), and 21.8% severe fatigue (> 35) (Fig. 2b). State fatigue, assessed with the NRS (0–10), increased from 3.79 (SD = 2.07) once at the start of the workday to 5.69 (SD = 1.87) at the end of the workday, with a mean change of 1.90 points (SD = 2.30).

Participants worked an average of 35.1 h per week (SD = 8.7). Most reported their work as mentally demanding (75.2%), with fewer describing it as physically demanding (6.9%) or both (17.8%). In total, 22.8% combined paid work with study obligations. Absence due to illness was reported by 75.2% in the past year, with an average of 2.1 sick days (SD = 2.4). Job characteristics were assessed using the Job Content Questionnaire. Compared to Dutch norm values, participants scored higher on skill discretion and decision authority, especially men. Psychological job demands were comparable to norms, while physical demands and job insecurity were slightly elevated. Supervisor support was higher among men, whereas coworker support was similar for both men and women. Full Job Content Questionnaire results are presented in Online Resource 3 and 4.

### Change in Cognitive Performance (Test-Level)

**Fig. 3:**
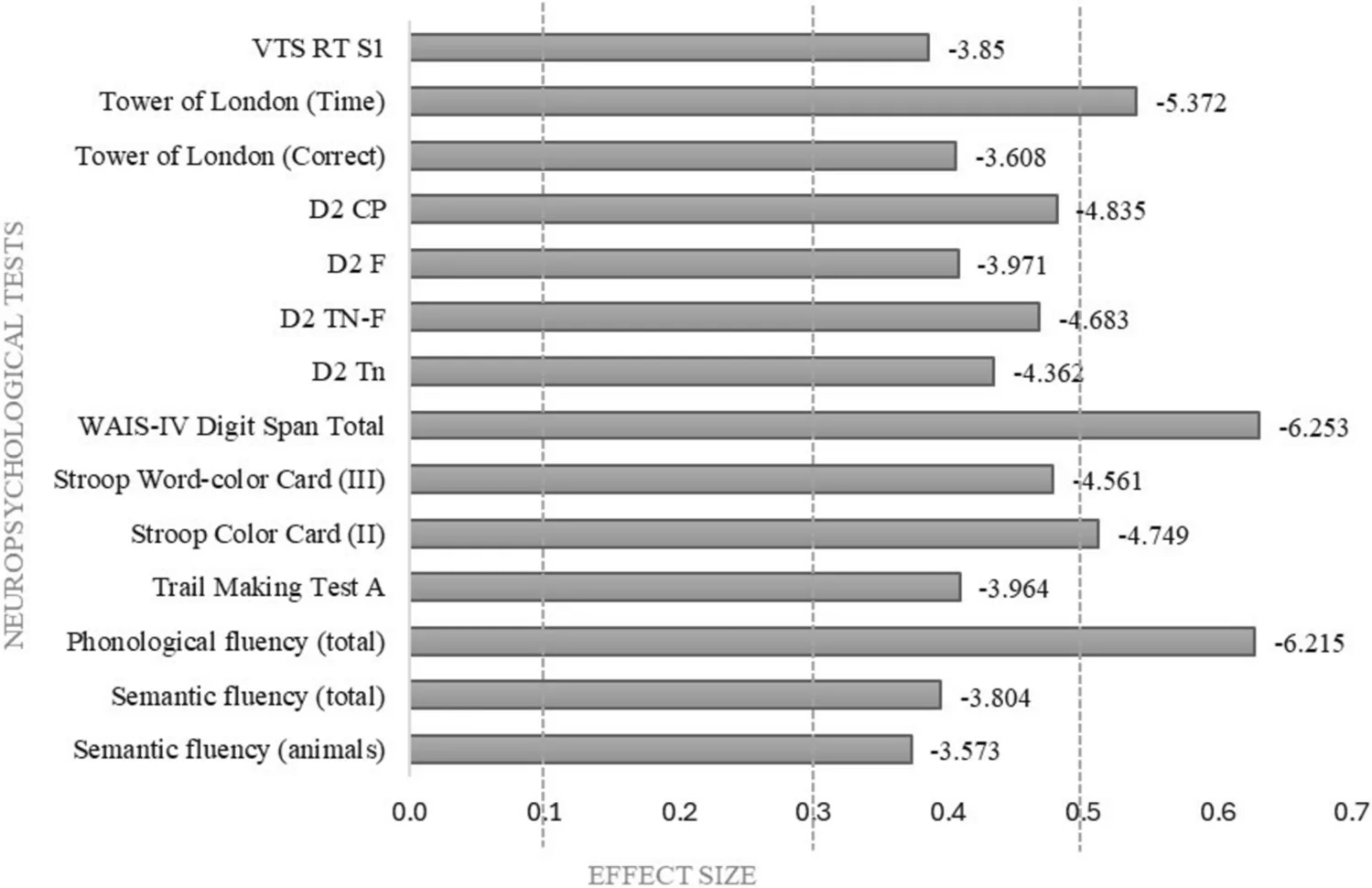
Effect sizes and Z-scores of significant pre–post-cognitive test changes. **Note:** Bars represent Wilcoxon Z-scores; corresponding standardized effect sizes $\left(r=\frac{z}{\sqrt{N}}\right)$ were derived from these tests. Dashed lines mark thresholds for small (r ≥ 0.1), medium (r ≥ 0.3), and large (r ≥ 0.5) effects. Negative Z-values reflect better post-work performance

Nineteen of 30 cognitive test scores showed a statistically significant change at the nominal level (p < 0.05), of which 14 remained significant after Bonferroni correction (adjusted α = 0.00167). As shown in Fig. 3, most significant changes reflected improved performance after work. The Wilcoxon Z-statistics for these tests ranged from 3.57 to 6.25, corresponding to standardized effect sizes (r) between 0.30 and 0.65. Full results, including Z-values and effect sizes, are presented in Online Resource 5. Reaction time on the VTS S1 task was the only outcome to show a significant decline, reflected by longer post-work response times (Z = –3.85, p < 0.001, r = 0.39), indicating slower performance after work.

Lastly, subjective fatigue also increased significantly across the workday, with higher NRS scores in the afternoon (M = 5.7, SD = 1.9) than in the morning (M = 3.8, SD = 2.1; *Z* = − 6.336, *p* < 0.001; Online Resource 6).

### Change in Cognitive Performance (Domain-Level)

**Fig. 4:**
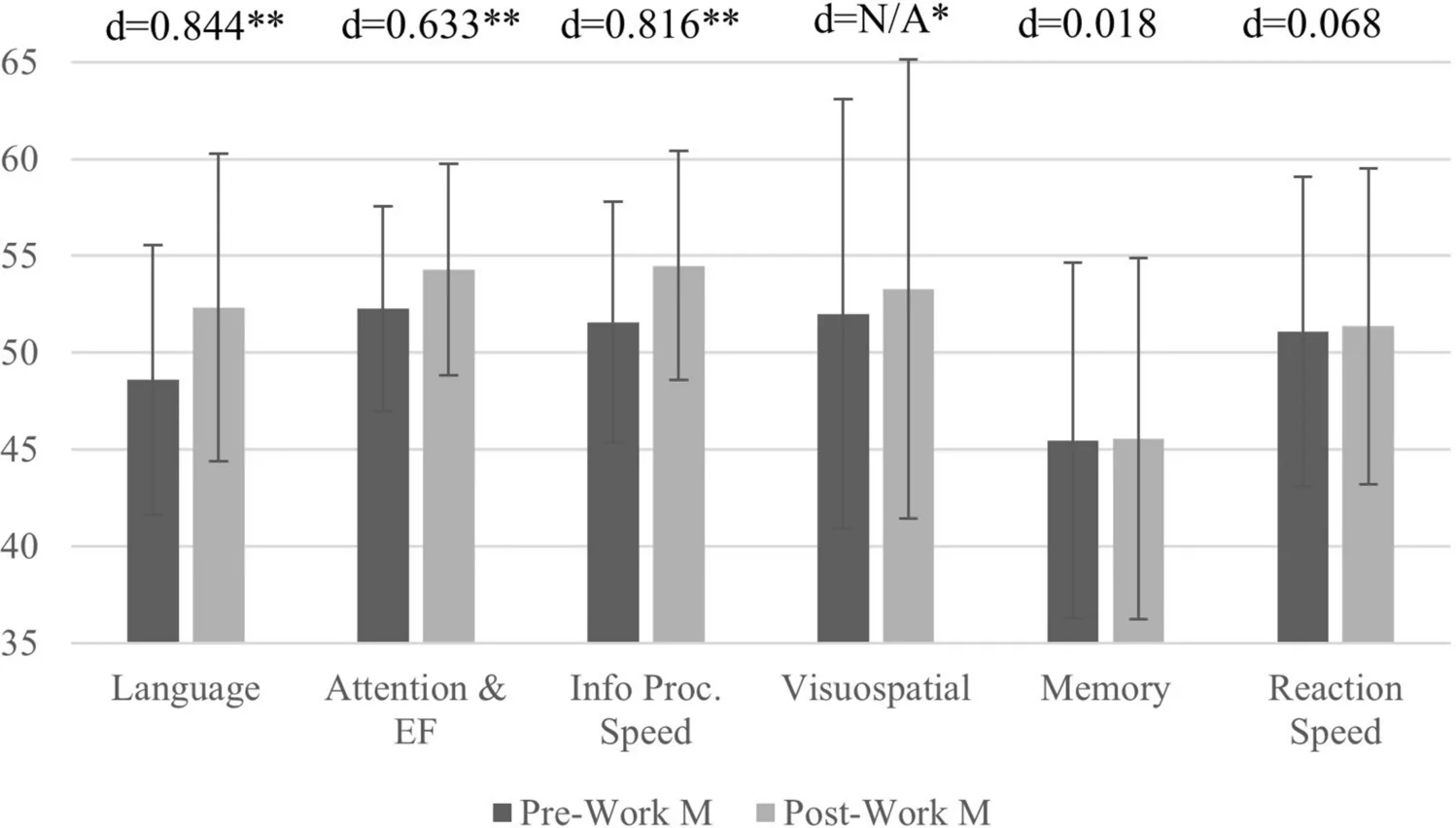
Mean composite *T*-scores for six cognitive domains pre- and post-work. Error bars indicate ± 1 standard deviation. Cohen’s *d* effect sizes are displayed above each pair of bars (*p* < .05, *p* < .01). Positive *d*-values denote higher post-work performance

Composite T-scores were calculated for six cognitive domains. Paired-samples *t*-tests showed significant improvements from pre- to post-work (T0 to T1) in language (t(100) = 8.48, p < 0.001, d = 0.844), attention and executive functioning (t(100) = 6.36, p < 0.001, d = 0.633), information processing speed (t(100) = 8.20, p < 0.001, d = 0.816). Visuospatial abilities revealed a small but statistically significant afternoon improvement (Z = 2.46, p = 0.014). In contrast, memory (t(100) = 0.19, p = 0.853, d = 0.018) and reaction speed (t(99) = 0.68, p = 0.496, d = 0.068) remained stable. See Fig. 4 and Online Resource 6.

### Correlations Between Fatigue and Performance

Spearman’s rank correlation was used to examine associations between within-day change scores (T1–T0) in cognitive domains and fatigue measures. Subjective trait fatigue, measured by the CIS total score and the CIS subjective fatigue subscale, showed significant correlations with language performance (*p* = –0.341, p < 0.001 and *p* = –0.317, p = 0.001, respectively), indicating that participants who reported higher fatigue levels demonstrated smaller gains or larger declines in language performance over the day. Post-work state fatigue (NRS) was also significantly correlated with language performance (*p* = –0.220, p = 0.027). No significant associations were found between fatigue measures and change scores in memory, attention and executive functioning, information processing speed, reaction speed, or visuospatial abilities (all p > 0.05).

Regarding work-related factors, none of the Job Content Questionnaire scales showed significant correlations with any cognitive domain. Full correlation matrices are provided in Online Resource 7a and 7b.

### Regression Analyses

Multiple linear regression analyses were conducted to examine whether fatigue-related variables predicted changes in cognitive performance. For each cognitive domain, the difference score (T1–T0) served as the dependent variable, and three predictors were entered simultaneously: pre-work fatigue (NRS), subjective fatigue (CIS subscale), and total fatigue (CIS total score).

The regression model for language was statistically significant, F(3, 97) = 4.51, p = 0.005, with an explained variance of R^2^ = 0.122. None of the individual predictors in this model were statistically significant (all p > 0.05). The regression models for memory, attention and executive functions, information processing speed, reaction speed, and visuospatial abilities were not statistically significant (all p > 0.05). One predictor reached statistical significance in the model for information processing speed: CIS subjective fatigue (B = –0.145, SE = 0.069, β = –0.423, t = –2.09, p = 0.040). Full regression coefficients are provided in Table 2. Model fit statistics are reported in Online Resource 8.

**Table 2:** Regression analyses on the difference score of cognitive domains and fatigue

Cognitive domain	Predictor	B	SE	β	t	*p*
Language	NRS_pre-work	0.135	0.227	0.063	0.595	0.553
	CIS subjective fatigue	− 0.068	0.083	− 0.161	− 0.823	0.413
	CIS total	− 0.047	0.040	− 0.224	− 1.187	0.238
Memory	NRS_pre-work	0.182	0.317	0.065	0.576	0.566
	CIS subjective fatigue	0.092	0.115	0.166	0.802	0.424
	CIS total	− 0.052	0.055	− 0.189	− 0.943	0.348
Attention and executive functions	NRS_pre-work	0.121	0.175	0.078	0.690	0.492
	CIS subjective fatigue	− 0.051	0.064	− 0.166	− 0.798	0.427
	CIS total	0.018	0.031	0.120	0.596	0.553
Information processing speed	NRS_pre-work	0.171	0.191	0.099	0.895	0.373
	CIS subjective fatigue	− 0.145	0.069	− 0.423	− 2.086	0.040*
	CIS total	0.036	0.033	0.213	1.088	0.279
Reaction speed	NRS_pre-work	0.371	0.207	0.201	1.795	0.076
	CIS subjective fatigue	− 0.111	0.076	− 0.302	− 1.464	0.146
	CIS total	0.020	0.036	0.112	0.564	0.574
Visuospatial abilities	NRS_pre-work	− 0.175	0.287	− 0.068	− 0.608	0.544
	CIS subjective fatigue	0.049	0.104	0.097	0.470	0.640
	CIS total	0.017	0.050	0.069	0.347	0.729

**Note:** For each cognitive domain, the dependent variable was the within-day difference score (T1–T0). Predictors were entered simultaneously using the enter method. B = unstandardized regression coefficient; SE B = standard error; β = standardized regression coefficient; NRS = Numeric Rating Scale (0–10); CIS = Checklist Individual Strength. *p < 0.05

## Discussion

This proof-of-principle study investigated whether cognitive endurance can be objectively assessed in a healthy working population by examining changes in cognitive performance over a standard workday using repeated neuropsychological testing. We aimed to explore whether subtle within-day cognitive fluctuations could be detected under naturalistic conditions and whether these fluctuations were related to self-reported fatigue.

The findings demonstrate that cognitive performance improved or remained stable over the day in most domains. Specifically, participants showed significant gains in language, attention and executive functioning, processing speed, and visuospatial ability. Memory and reaction speed remained unchanged. These results confirm that healthy individuals are capable of sustaining cognitive performance throughout a typical workday. As participants did not complete a separate familiarization session, part of the observed improvement may reflect practice or adaptation effects. These effects are, however, inherent to repeated-measures designs and provide meaningful information about cognitive adaptability across the day.

The improvements are likely explained by practice effects. Repetition of cognitive tasks can enhance efficiency through procedural learning, task familiarity, and reduced cognitive interference [38–41]. Gains were most evident in domains known to benefit from repeated exposure, such as fluency and processing speed. Improvements in visuospatial ability may reflect visual-motor habituation. In contrast, the stability in memory and reaction speed may be due to reduced sensitivity to practice effects or subtle fatigue-related declines that remain undetected [13, 14].

These findings offer insight into the expected variability of test performance in cognitively healthy adults over a day. Although short-term within-day change is rarely addressed in neuropsychological assessment, our results suggest that modest performance gains, rather than declines, are typical in repeated testing across a workday, which is consistent with previous research on repeated measurements. This pattern may reflect short-term practice or adaptation effects, indicating that individuals can maintain or even improve cognitive efficiency despite prolonged task engagement. These findings underline the need to interpret within-day cognitive change carefully, particularly in clinical or rehabilitation samples where apparent stability or improvement may mask underlying fatigue effects. Clinicians and researchers should therefore consider retest and adaptation effects when evaluating cognitive endurance or recovery throughout the day, while recognizing that slight performance declines might warrant further investigation to determine whether they reflect reduced endurance or normal day-to-day variability.

At baseline, a substantial number of participants reported elevated or severe trait fatigue. Over the course of the workday, subjective fatigue increased significantly. While baseline and pre-work fatigue were not significantly associated with cognitive change, subjective fatigue was a significant predictor of change in information processing speed. This limited association is consistent with previous findings showing that perceived exhaustion does not always translate into measurable cognitive decline [6, 16, 42], and that subjective complaints often show weak or no correlation with objective performance, even in cognitively healthy populations [43]. This may be due in part to compensatory mechanisms, where individuals exert additional effort or use more efficient strategies to maintain task performance under fatigue [17, 44].

Regression analyses showed that the model predicting change in language performance was statistically significant (R^2^ = 0.122), although none of the individual fatigue predictors reached significance in that model. In the model for information processing speed, subjective fatigue (CIS subscale) was a significant predictor. For the remaining cognitive domains, regression models were not statistically significant, and fatigue predictors were not associated with performance change. This limited number of associations suggests that self-reported fatigue has only partial explanatory value for cognitive fluctuations over a workday. Other factors, such as resilience, task engagement, or psychological state, may play a more substantial role [15, 18].

These results have practical implications. In occupational and insurance medicine, complaints of fatigue are frequently used to justify limitations in work capacity. Our findings underscore the value of incorporating objective cognitive assessments into these evaluations. While subjective fatigue is a valid and important signal, it does not necessarily indicate cognitive dysfunction or decreased cognitive performance within-day. This discrepancy has also been observed in disability assessments, where reported symptoms, particularly in mental health contexts, do not always align with functional capacity as determined by more structured evaluations [45]. A combined approach that integrates subjective experiences with performance-based data may lead to more accurate and equitable judgments about work ability.

In contexts where return-to-work decisions rely on cognitive assessment, additional considerations are warranted. When assessing individuals with suspected cognitive impairment or fatigue-related limitations, standard neuropsychological protocols may require adaptation to prevent overburdening and misinterpretation. Clinicians should take potential practice and adaptation effects into account and interpret repeated testing results within the broader context of day-to-day variability. Incorporating validity or effort indicators is essential to ensure credible performance, particularly in high-stakes evaluations. Repeated assessments across a workday or multiple days could also provide a more ecologically valid representation of cognitive endurance and recovery. Integrating both subjective and objective measures, and interpreting them within the context of daily functioning, will be critical for guiding rehabilitation strategies and return-to-work planning. Our findings raise questions about the use of the CIS in non-clinical, working populations. Although the instrument effectively captures subjective fatigue, nearly half of our participants scored in the increased or severe range on the fatigue subscale, despite being generally healthy and employed. This suggests that clinical cut-offs may not be fully appropriate in occupational settings and that interpretation may require adjustment or context-specific norms [21].

Several limitations should be noted. The sample was predominantly highly educated and employed in mentally demanding roles, which may limit generalizability. Moreover, the study defined “healthy working adults” from a functional rather than biomedical perspective, reflecting participants’ ability to work despite minor or stable health conditions. While this enhances ecological validity, it may limit comparability with studies using stricter health criteria. Sensitivity to fatigue may vary across occupations, and future research should include more diverse and physically oriented work settings. Furthermore, we did not assess psychological traits such as coping style or mood, which may influence both fatigue and cognitive performance [46]. Lastly, the study covered only a single workday. Multi-day assessments or testing during periods of higher workload may offer deeper insight into true cognitive endurance under sustained occupational demands [12, 47]. Data collection occurred over two years without standardization for weekday or season, which may have introduced natural variation in workload or fatigue cycles. However, this variability reflects real-world conditions and is unlikely to have systematically influenced the within-subject comparisons.

In conclusion, this study shows that cognitive performance in healthy working adults is generally stable or improves over the course of a regular workday, despite increasing subjective fatigue. It also emphasizes the importance of interpreting self-reported complaints in the context of objective performance. Future research should expand on these findings by including clinical groups, extending assessment periods, and exploring more ecologically valid and individualized approaches to cognitive endurance measurement.

## Supplementary Information

Below is the link to the electronic supplementary material.

Supplementary file1 (DOCX 61 KB)

## Data Availability

The data generated and/or analyzed during the current study are available from the corresponding author on reasonable request.
