# Supplementary material for: Toward Measuring Cognitive Endurance Capacity: A Proof-of-Principle Study in a Healthy Population
Source: J Occup Rehabil. 2026 Jan 13;36(4):1210–20. doi: 10.1007/s10926-025-10346-8 (PMC13759664; doi:10.1007/s10926-025-10346-8)
Supplement: Supplementary file 1 [file 10926_2025_10346_MOESM1_ESM.docx]

**Supplementary Information, Online Resource 1-8**

**Article Title:**	Towards Measuring Cognitive Endurance Capacity: A Proof-of-Principle Study in a Healthy Population

**Journal:**	Journal of Occupational Rehabilitation

**Authors:**	Fabienne I.M. van Vliet, Birgit H.P.M. Donker-Cools, Gert J. Geurtsen, Rudolf W.H.M. Ponds, Frederieke G. Schaafsma

**E-mail:**	f.i.vanvliet@amsterdamumc.nl

**Online Resource 1.** *Demographics of 101 healthy workers (Age M: 35.71 SD 13.56)*

| **Characteristics of the sample (101)** | | **N (%)** |  |
| --- | --- | --- | --- |
| Sex |  |  |  |
|  | Male | 44 (43.6) |  |
|  | Female | 57 (56.4) |  |
| Education Level |  |  |  |
|  | Low educational level (≤4) | 4 (4) |  |
|  | Middle educational level (5) | 15 (14.9) |  |
|  | High educational level (6-7) | 82 (81.2) |  |
|  |  | **N (%)** | **N Male/Female (%)** |
| Somatic disorder | Yes | 10 (9.9) | 3/7 (3/6.9) |
| DSM diagnosis | Yes | 16 (15.8) | 8/8 (7.9/7.9) |
| Medication | Yes | 23 (23.8) | 10/13 (9.9/12.9) |

Note. Education levels are based on the Dutch Verhage scale (1–7), with ≤4 classified as low, 5 as middle, and 6–7 as high. Values are presented for the total sample (N = 101). The male/female distribution is shown for somatic disorders, DSM diagnoses, and medication use.

**Online Resource 2.** *General Health Questionnaire-12.*

| **General Health Questionnaire-12** | | **N (%)** |
| --- | --- | --- |
| Score < 2 |  | 67 (66.3) |
| Score ≥ 2 |  | 34 (33.7) |

Note. GHQ-12 total scores range from 0 to 12. Higher scores indicate poorer general mental health. Scores of 2 or higher suggest reduced psychological well-being.

**Online Resource 3.** *Job descriptive and work characteristics of participants.*

| **Job characteristics** | | **N (%)** |
| --- | --- | --- |
| Working situation |  |  |
|  | Working | 78 (77.2) |
|  | Studying and working | 23 (22.8) |
| Type of wok |  |  |
|  | Physical demanding | 7 (6.9) |
|  | Mentally demanding | 76 (75.2) |
|  | Both | 18 (17.8) |
| Absence history in past year |  |  |
|  | No | 25 (24.8) |
|  | Yes | 76 (75.25) |
| Number of days sick during last absence | M 2.12 (SD 2.4) |  |
| Average working hours a week | M 35.13 (SD 8.68) |  |

Note. Values represent the number and percentage of participants per category. M = mean; SD = standard deviation.

**Online Resource 4.** *Job Content Questionnaire descriptive (JCQ)*

| **Scale** | **Range score** | **Females Normvalues** | **Sample Females** | **Males Normvalues** | **Sample Males** |
| --- | --- | --- | --- | --- | --- |
|  |  | **M (SD)** | **M (SD)** | **M (SD)** | **M (SD)** |
| Skill discretion | 12-48 | 31.7 (5.4) | 35.82 (6.03) | 33.8 (5.3) | 38.14 (5.32) |
| Decision authority | 8-48 | 31.4 (7.2) | 36.98 (7.87) | 33.3 (6.8) | 38.91 (7.64) |
| Psychological demand | 12-36 | 32.1 (5.1) | 31.58 (5.94) | 31.6 (5.2) | 29.91 (6.11) |
| Physical demand | 3-12 | 2.6 (0.9) | 7.96 (3.34) | 2.7 (0.9) | 7.73 (3.16) |
| Job insecurity | 3-12 | 3.1 (0.9) | 4.37 (0.67) | 3.3 (1.0) | 4.50 (0.70) |
| Supervisor support | 4-16 | 11.0 (2.2) | 11.07 (2.40) | 10.9 (2.2) | 12.02 (2.25) |
| Coworker support | 4-16 | 12.2 (1.7) | 12.39 (1.86) | 12.0 (1.6) | 12.52 (1.39) |
| Decision latitude* | 20-96 | 63.0 (11.2) | 72.81 (12.52) | 67.3 (10.7) | 77.05 (11.31) |

Note. Higher scores on Skill Discretion, Decision Authority, and Social Support indicate more favorable job characteristics. Higher scores on Psychological Demand, Physical Demand, and Job Insecurity indicate higher work strain.

*Decision Latitude represents the combined score of Skill Discretion and Decision Authority

**Online Resource 5**. *Results of Wilcoxon Signed-Rank Tests Comparing Pre- and Post-Work Cognitive Performance (Bonferroni-Corrected Significance Threshold α = 0.00167)*

| **Variables/(Sub)tests** | **Pre-work** | | **Post-work** | | **Negative Ranksⁱ** | | | **Positive Ranksⁱⁱ** | | | **Test statistics** | | | **Bonferroni correction** | | **Effect size** | |
| --- | --- | --- | --- | --- | --- | --- | --- | --- | --- | --- | --- | --- | --- | --- | --- | --- | --- |
|  | M (SD) | Median | M (SD) | Median | N | Mean ranks | Sum of ranks | N | Mean ranks | Sum of ranks | Tielsⁱⁱⁱ | Z-value | *p*-value | | α =0.00167 Y/N | | *r* |
| Semantic Fluency  Animals | 28.1 (6.5) | 29 | 29.9 (6.4) | 30 | 57 | 53.57 | 3053.50 | 35 | 34.99 | 1224.50 | 9 | -3.573 | <0.001 | | Y | | 0.373 |
| Semantic Fluency Occupations | 21.2 (4.9) | 20 | 22.1 (6.0) | 22 | 50 | 51.13 | 2556.50 | 42 | 40.99 | 1721.50 | 9 | -1.633 | 0.103 | | N | | N/A |
| Semantic Fluency  Total score | 49.1 (10.2) | 49 | 51.6 (11.3) | 51 | 60 | 52.95 | 3177.00 | 33 | 36.18 | 1194.00 | 8 | -3.804 | <0.001 | | Y | | 0.395 |
| Phonological Fluency  Total score | 37.6 (9.4) | 37 | 43.9 (11.4) | 44 | 76 | 54.98 | 4178.50 | 22 | 30.57 | 672.50 | 3 | -6.215 | <0.001 | | Y | | 0.628 |
| AVLT  Immediate recall | 49.9 (11.1) | 51 | 51.3 (11.1) | 51 | 56 | 51.04 | 2858.00 | 40 | 44.95 | 1798.00 | 5 | -1.939 | 0.053 | | N | | N/A |
| AVLT  Delayed recall | 11.0 (3.3) | 12 | 11.0 (3.2) | 12 | 37 | 38.80 | 1435.50 | 38 | 37.22 | 1414.50 | 26 | -0.056 | 0.955 | | N | | N/A |
| RBMT Stories  Immediate recall | 20.0 (6.6) | 19.5 | 19.5 (6.1) | 19 | 40 | 50.19 | 2007.50 | 57 | 48.17 | 2745.50 | 4 | -1.329 | 0.184 | | N | | N/A |
| RBMT Stories   Delayed recall | 16.3 (6.8) | 15.5 | 16.2 (6.4) | 16 | 44 | 49.15 | 2162.50 | 51 | 47.01 | 2397.50 | 4 | -0.437 | 0.662 | | N | | N/A |
| WAIS-IV Digit Span Total | 28.2 (4.6) | 28 | 28.6 (5.3) | 28.0 | 47 | 48.44 | 2276.50 | 43 | 42.29 | 1818.50 | 11 | -0.926 | 0.354 | | N | | N/A |
| Trail making test  Part A | 24.7 (9.4) | 23 | 21.8 (7.6) | 21 | 35 | 33.81 | 1182.50 | 59 | 55.62 | 3281.50 | 7 | -3.964 | <0.001 | | Y | | 0.409 |
| Trail making test  Part B | 53.1 (17.4) | 49 | 48.6 (14.0) | 48 | 43 | 36.52 | 1570.50 | 53 | 58.22 | 3085.50 | 5 | -2.770 | 0.006 | | N | | N/A |
| Stroop color word test   Stroop word card (I) | 39.3 (6.1) | 38 | 38.8 (6.2) | 38 | 36 | 34.67 | 1248.00 | 41 | 42.80 | 1755.00 | 23 | -1.329 | 0.184 | | N | | N/A |
| Stroop color word test   Stroop color card (II) | 53.7 (10.4) | 52 | 50.9 (8.1) | 50 | 23 | 33.76 | 776.50 | 63 | 47.06 | 2964.50 | 14 | -4.749 | <0.001 | | Y | | 0.512 |
| Stroop color word test   Stroop word color (III) | 80.1 (19.1) | 78 | 76.1 (16.7) | 74 | 28 | 33.77 | 945.50 | 63 | 51.44 | 3240.50 | 9 | -4.561 | <0.001 | | Y | | 0.478 |
| WAIS-IV  Symbol subs coding | 74.3 (13.6) | 74 | 79.3 (15.6) | 77 | 79 | 53.02 | 4188.50 | 19 | 34.87 | 662.50 | 3 | -6.253 | <0.001 | | Y | | 0.632 |
| D2 Tn | 508.7 (81.6) | 522 | 527.7 (79.2) | 540 | 71 | 54.41 | 3863.00 | 30 | 42.93 | 1288.00 | 0 | -4.362 | <0.001 | | Y | | 0.434 |
| D2 Tn-F | 488.4 (90.4) | 505 | 505.6 (90.3) | 514 | 74 | 52.52 | 3886.50 | 26 | 44.75 | 1163.50 | 1 | -4.682 | <0.001 | | Y | | 0.468 |
| D2 F | 21.2 (24.8) | 14 | 16.4 (22.9) | 11 | 26 | 46.58 | 1211.00 | 69 | 48.54 | 3349.00 | 6 | -3.971 | <0.001 | | Y | | 0.407 |
| D2 VT | 11.8 (6.0) | 11 | 10.7 (4.6) | 10 | 42 | 40.00 | 1680.00 | 48 | 50.31 | 2415.00 | 11 | -1.482 | 0.138 | | N | | N/A |
| D2 CP | 197.3 (50.3) | 199 | 209.5 (50.6) | 211 | 76 | 52.66 | 4002.50 | 25 | 45.94 | 1148.50 | 0 | -4.835 | <0.001 | | Y | | 0.481 |
| Tower of London  Total correct | 4.5 (2.3) | 5 | 5.3 (2.5) | 6 | 60 | 38.48 | 2308.50 | 19 | 44.82 | 851.50 | 21 | -3.608 | <0.001 | | Y | | 0.406 |
| Tower of London  Total move score | 30.4 (17.8) | 29 | 24.2 (18.3) | 20 | 37 | 40.49 | 1498.00 | 60 | 54.25 | 3255.00 | 2 | -3.162 | 0.002 | | N | | N/A |
| Tower of London  Problem-solving time | 250.1 (91.3) | 243 | 201.6 (69.6) | 193.5 | 28 | 33.43 | 936.00 | 71 | 56.54 | 4014.00 | 0 | -5.372 | <0.001 | | Y | | 0.540 |
| VTSRT S1  Reaction time | 297.7 (47.5) | 299.5 | 312.7 (53.9) | 306 | 66 | 55.22 | 3644.50 | 34 | 41.34 | 1405.50 | 0 | -3.850 | <0.001 | | Y | | 0.385 |
| VTSRT S1  Movement time | 151.9 (49.6) | 142.5 | 150.2 (48.8) | 143 | 44 | 49.48 | 2177.00 | 51 | 46.73 | 2383 | 5 | -0.382 | 0.702 | | N | | N/A |
| VTSRT S1 Errors | 0.4 (0.9) | 0 | 0.4 (0.9) | 0 | 15 | 15.63 | 234.50 | 16 | 16.34 | 261.50 | 69 | -0.285 | 0.775 | | N | | N/A |
| VT RT S3  Reaction time | 457.2 (94.2) | 454 | 448.1 (84.2) | 449.5 | 39 | 46.28 | 1805.00 | 58 | 50.83 | 2948.00 | 1 | -2.056 | 0.040 | | N | | N/A |
| VT RT S3  Movement time | 166,2 (77,5) | 153,5 | 155.1 (49.4) | 150 | 34 | 50.16 | 1705.50 | 61 | 46.80 | 2854.50 | 3 | -2.133 | 0.033 | | N | | N/A |
| VTSRT S3 Errors | 0,5 (1,3) | 0 | 0,3 (0,6) | 0 | 13 | 13.00 | 169.00 | 16 | 16.63 | 266.00 | 69 | -1.139 | 0.255 | | N | | N/A |
| GIT2 Perceptual Puzzle | 13,0 (4,0) | 14 | 13,4 (4,3) | 14 | 42 | 39.96 | 1678.50 | 29 | 30.26 | 877.50 | 30 | -2.343 | 0.019 | | N | | N/A |
| NRS | 3,8 (2,1) | 4 | 5,7 (1,9) | 6 | 77 | 48.79 | 3756.50 | 15 | 34.77 | 521.50 | 9 | -6.336 | <0.001 | | N/A | | - |

Note.

ⁱ Post-work <Pre-work, Negative ranks: number of participants who performed worse post-work than pre-work

ⁱⁱ Post-work > Pre-work, Positive ranks: number of participants who performed better post-work than pre-work

ⁱⁱⁱ Post work= Pre-work, Ties: number of participants with identical scores pre- and post-work

Negative ranks indicate better post-work performance; positive ranks indicate worse post-work performance. Negative Z-values thus reflect improvement.*Y = Significant difference based on Bonferroni-corrected threshold (p < 0.00167) / N = Not significant after Bonferroni correction.*

*Effect sizes (*r*) were calculated using the formula* $r=\frac{z}{\sqrt{N}}$*, where* Z *is the Wilcoxon test statistic and* N *the number of non-tied observations.*

*AVLT = Auditory Verbal Learning Test, RBMT = Rivermead Behavioural Memory Test, WAIS-IV = Wechsler Adult Intelligence Scale – Fourth Edition, D2 = D2 Test of Attention, Tn = Total number, Tn-F = Total number minus errors, F = Number of errors, VT = Variability in performance (Fluctuation Index), CP = Concentration Performance, VTSRT = Vienna Test System Reaction Test, GIT2 = Groninger Intelligence Test–2, NRS = Numeric Rating Scale (state fatigue score)*

**Online Resource 6.** *Differences Between Morning and Afternoon Cognitive Performance by Domain, Based on Averaged T-Scores (Pre- and Post-Work Comparison)*

| **Cognitive Domain** | **Pre-work**  **M (SD)** | **Post-work**  **M (SD)** | **Difference score** | **t/Z (df)** | ***p*** | **Effect size (d)** |
| --- | --- | --- | --- | --- | --- | --- |
| Language | 48.59 (6.96) | 52.32 (7.93) | 3.73 | 8.48 (100) | <0.001** | 0.844 |
| Attention & Executive Functions | 52.26 (5.30) | 54.29 (5.45) | 2.03 | 6.36 (100) | <0.001** | 0.633 |
| Information Processing Speed | 51.56 (6.23) | 54.48 (5.92) | 2.92 | 8.20 (100) | <0.001** | 0.816 |
| Visuospatial | 51.99 (11.08) | 53.28 (11.83) | 1.29 | 2.46 | 0.014* | N/A |
| Memory | 45.45 (9.19) | 45.56 (9.32) | 0.11 | 0.19 (100) | 0.853 | 0.018 |
| Reaction Speed | 51.08 (7.98) | 51.35 (8.14) | 0.26 | 0.68 (99) | 0.496 | 0.068 |

Note. Paired-samples t-tests were used for all domains except Visuospatial Abilities, which violated normality assumptions and was therefore tested using a Wilcoxon Signed-Rank Test.

T-scores were averaged per domain. Two-sided p-values are reported: p < 0.05 = significant (); p < 0.001 = highly significant (**). Effect size d represents Cohen’s d (0.2 = small, 0.5 = medium, 0.8 = large). N/A = not applicable.*

**Online Resource 7a**. Correlation analyses on the difference score of cognitive domains and fatigue-related measures

| **Variable** | **Spearman’s rho (ρ)** | | | | | | | | | | |
| --- | --- | --- | --- | --- | --- | --- | --- | --- | --- | --- | --- |
|  | 1 | 2 | 3 | 4 | 5 | 6 | 7 | 8 | 9 | 10 | 11 |
| 1 Language | 1 |  |  |  |  |  |  |  |  |  |  |
| 2 Memory | -0.007 | 1 |  |  |  |  |  |  |  |  |  |
| 3 Attention & Executive Functions | 0.088 | 0.151 | 1 |  |  |  |  |  |  |  |  |
| 4 Information Processing Speed | 0.165 | 0.022 | 0.282** | 1 |  |  |  |  |  |  |  |
| 5 Reaction Speed | -0.033 | 0.131 | 0.279** | 0.197* | 1 |  |  |  |  |  |  |
| 6 Visuospatial Abilities | 0.046 | -0.047 | 0.165 | 0 | -0.103 | 1 |  |  |  |  |  |
| 7 NRS pre-work | -0.082 | 0.044 | 0.042 | -0.023 | 0.064 | 0.002 | 1 |  |  |  |  |
| 8 NRS post-work | -0.220* | 0.035 | -0.169 | -0.145 | -0.008 | 0.058 | 0.288** | 1 |  |  |  |
| 9 NRS difference | -0.138 | 0.029 | -0.155 | -0.146 | -0.089 | 0.09 | -0.639** | 0.497** | 1 |  |  |
| 10 CIS subjective fatigue | -0.317** | 0.038 | -0.038 | -0.184 | -0.127 | 0.164 | 0.463** | 0.342** | -0.09 | 1 |  |
| 11 CIS total score | -0.341** | -0.027 | 0.018 | -0.107 | -0.089 | 0.137 | 0.400** | 0.317** | -0.064 | 0.861** | 1 |

Note. Spearman’s rho (ρ) coefficients represent the strength and direction of associations between cognitive change scores and fatigue measures. Negative values indicate that higher fatigue is associated with lower cognitive performance.

*Correlation is significant at the 0.05 level (2-tailed); ***Correlation is significant at the 0.01 level (2-tailed).*

*NRS = Numeric Rating Scale; CIS = Checklist Individual Strength.*

**Online Resource 7b.** *Correlation analyses on the difference score of cognitive domains and work related factors (JCQ)*

| **Variable** | **Spearman’s rho (ρ)** | | | | | | | | | | | | |
| --- | --- | --- | --- | --- | --- | --- | --- | --- | --- | --- | --- | --- | --- |
|  | 1 | 2 | 3 | 4 | 5 | 6 | 7 | 8 | 9 | 10 | 11 | 12 | 13 |
| 1 Language | 1 |  |  |  |  |  |  |  |  |  |  |  |  |
| 2 Memory | -0.007 | 1 |  |  |  |  |  |  |  |  |  |  |  |
| 3 Attention & Executive Funct. | 0.088 | 0.151 | 1 |  |  |  |  |  |  |  |  |  |  |
| 4 Info processing Speed | 0.165 | 0.022 | 0.282** | 1 |  |  |  |  |  |  |  |  |  |
| 5 Reaction Speed | -0.033 | 0.131 | 0.279** | 0.197* | 1 |  |  |  |  |  |  |  |  |
| 6 Visuospatial Abilities | 0.046 | -0.047 | 0.165 | 0.000 | -0.103 | 1 |  |  |  |  |  |  |  |
| 7. JCQ_Skill_discretion | 0.133 | 0.158 | 0.130 | -0.078 | -0.009 | 0.038 | 1 |  |  |  |  |  |  |
| 8. JCQ_Decision_autohority | -0.017 | 0.066 | 0.013 | -0.141 | -0.012 | 0.103 | 0.546** | 1 |  |  |  |  |  |
| 9. JCQ_Psychological_demand | 0.064 | -0.054 | -0.032 | -0.089 | -0.059 | -0.088 | 0.149 | 0,030 | 1 |  |  |  |  |
| 10. JCQ_Physical_demand | -0.165 | -0.016 | -0.065 | 0.021 | -0.139 | -0.059 | -0.058 | -0.095 | 0.286** | 1 |  |  |  |
| 11. JCQ_Job_insecurity | 0.019 | -0.100 | 0.054 | 0.020 | 0.044 | 0.045 | 0.182 | 0.385** | 0.057 | -0.161 | 1 |  |  |
| 12. JCQ_Supervisor_support | -0.165 | 0.102 | -0.120 | -0.088 | 0.057 | -0.067 | 0.415^**^ | 0.369^**^ | -0.198^*^ | -0.183 | 0.062 | 1 |  |
| 13. JCQ_Coworker_support | 0.041 | 0.083 | -0.031 | 0.033 | 0.064 | -0.016 | 0.355** | 0.268** | -0.021 | -0.194 | 0.058 | 0.458** | 1 |

Note. Spearman’s rho (ρ) coefficients represent the strength and direction of associations between cognitive change scores and job-related factors.

*Correlation is significant at the 0.05 level (2-tailed); **Correlation is significant at the 0.01 level (2-tailed).

**Online Resource 8.** *Model fit statistics for regression analyses predicting within-day cognitive change*

| **Cognitive Domain** | **R²** | **Adj. R²** | **F(df1,df2)** | ***p* (model)** |
| --- | --- | --- | --- | --- |
| Language | 0.122 | 0.095 | F(3,97) = 4.51 | 0.005* |
| Memory | 0.014 | -0.017 | F(3,97) = 0.45 | 0.721 |
| Attention and Executive Functions | 0.009 | -0.022 | F(3,97) = 0.30 | 0.827 |
| Information Processing Speed | 0.057 | 0.027 | F(3,97) = 1.94 | 0.128 |
| Reaction Speed | 0.048 | 0.018 | F(3,96) = 1.60 | 0.194 |
| Visuospatial Abilities | 0.021 | -0.009 | F(3,97) = 0.69 | 0.561 |

Note. R² and adjusted R² represent the proportion of variance in within-day cognitive performance change (T1–T0) explained by the fatigue-related predictors. p-values indicate the statistical significance of the model coefficients. *p < 0.05.* Positive change scores indicate improvement.
